# Standards: New Yardstick for Medicinal Plant Harvests

**DOI:** 10.1289/ehp.116-a21a

**Published:** 2008-01

**Authors:** David A. Taylor

Every year more than 400,000 tons of medicinal and aromatic plants from approximately 3,000 species are traded internationally, according to TRAFFIC, a nonprofit watchdog group that monitors commerce in natural products. (Up to 70,000 species are used medicinally worldwide, most of them locally.) Such a growth in demand for these plants threatens natural resources, since about 80% of commercially traded species are gathered from the wild, according to the World Conservation Union (IUCN). In February 2007, several groups concerned about potential adverse effects of this rise on plant habitats announced an international standard designed to preserve nature’s medicine chest for future generations. A year later, the standard appears to be bearing fruit.

The IUCN Medicinal Plant Specialist Group, IUCN Canada, the German Federal Agency for Nature Conservation, WWF Germany, and TRAFFIC proposed the standard and coordinated several rounds of international vetting in 2005 and 2006. The new International Standard for Sustainable Wild Collection of Medicinal and Aromatic Plants (ISSC-MAP) is intended to balance the needs of people whose traditions and livelihood depend on these species with the plants’ long-term survival in their native habitats.

The new standard is based on six principles related to maintaining wild resources, preventing negative environmental impacts, respecting customary rights (for example, of indigenous populations), and exercising responsible management and business practices. Plant scientists also drew on earlier guidelines both for the conservation of medicinal plants and for good agricultural and collection practices. “We did not want to reinvent the wheel,” says Susanne Honnef, TRAFFIC medicinal plant officer with WWF Germany, “so the standard builds on existing frameworks.”

The new standard involves all actors along the supply chain—from wild plant harvesters to sellers—in a process for determining how to sustainably conduct harvests and trade, says Honnef. The standard also outlines practices for monitoring the impact of harvests over time.

Honnef says the standard will protect important natural resources. As the benefits of sustainable use become more broadly recognized, harvesters will be encouraged to protect the ecosystems that support their livelihoods. And government agencies will have tools for defining benchmarks in a trade that is often informal and that falls through the cracks between groups that manage agriculture and forestry.

The standard was tested in preliminary trials undertaken in six countries. Over 6 months in 2007, for example, 2 Indian communities used the standard to gauge population health of 6 commercially traded species, says Giridhar Kinhal, special projects coordinator for Foundation for Revitalisation of Local Health Traditions, a nonprofit scientific and research organization in Bangalore. Based on that trial, says Kinhal, the communities saw improved regeneration of the studied plant populations, but also reported the need for further guidance in assimilating these outcomes into resource management. Next comes a 2-year implementation phase at sites in Asia, Africa, southeast Europe, and South America.

Danna Leaman, chair of the IUCN Medicinal Plant Specialist Group and a member of the advisory group that guided the development of ISSC-MAP, says, “A concrete activity like this is a real step forward” for the IUCN, which has worked for years to engage industry in biodiversity protection. Indeed, ISSC-MAP goes even further than current guidance such as Fair Trade and Organic certification.

For example, it has been up to individual inspectors to determine whether a wild collection operation meets the requirements for Organic certification. Josef Brinckmann, vice president for research with manufacturer Traditional Medicinals, says that although the wild botanicals they use qualify for Organic certification, some sites will need further work to conform with all 6 principles of the ISSC-MAP. “Many of these certified Organic wild collection sites would need a few years to make the necessary changes for conformance with the ISSC-MAP standard,” he says. Yet, Brinckmann adds, the extra work will be worthwhile if compliance with the standard can help a company demonstrate unequivocably that its operations help maintain the botanical resource.

Brinckmann points to Asia and Europe as places where the standard may first have a significant impact in alleviating intense harvest pressures. “China and India are the two largest producers and exporters of medicinal plants in the world,” he notes. Southeastern European countries and Russia are also important in the world market.

